# Bucindolol Modulates Cardiac Remodeling by Attenuating Oxidative Stress in H9c2 Cardiac Cells Exposed to Norepinephrine

**DOI:** 10.1155/2019/6325424

**Published:** 2019-07-10

**Authors:** Bruna Gazzi de Lima-Seolin, Ashley Nemec-Bakk, Heidi Forsyth, Stefanie Kirk, Alex Sander da Rosa Araujo, Paulo Cavalheiro Schenkel, Adriane Belló-Klein, Neelam Khaper

**Affiliations:** ^1^Laboratory of Cardiovascular Physiology and Reactive Oxygen Species, Institute of Basic Health Science (ICBS), Universidade Federal do Rio Grande do Sul (UFRGS), Porto Alegre 90050-170, Brazil; ^2^Medical Sciences Division, Northern Ontario School of Medicine (NOSM), Lakehead University, Thunder Bay, ON P7B 5E1, Canada; ^3^Department of Biology, Lakehead University, Thunder Bay, ON P7B 5E1, Canada

## Abstract

The increased circulation of norepinephrine, found in the diseased heart as a result of sympathetic nervous system overactivation, is responsible for its cardiotoxic effects including pathological hypertrophy, cell death, and oxidative stress. Bucindolol is a third generation adrenergic blocker, which acts on the *β*1 and *β*2 receptors, and has additional *α*1 antagonist activity. Thus, the aim of this study was to investigate the action of bucindolol on oxidative stress, hypertrophy, cell survival, and cell death signaling pathways in H9c2 cardiac cells exposed to norepinephrine. H9c2 cells were incubated with 10 *μ*M norepinephrine for 24 h in the presence or absence of bucindolol (10 *μ*M) treatment for 8 h. Western blot was used to determine the expression of proteins for hypertrophy/survival and death signaling pathways. Flow cytometry was used to assess cell death via caspase-3/7 activity and propidium iodide and reactive oxygen species via measuring the fluorescence of CM-H_2_DCFDA. Norepinephrine exposure resulted in an increase in oxidative stress as well as cell death. This was accompanied by an increased protein expression of LC3B-II/I. The protein kinase B/mammalian target of the rapamycin (Akt/mTOR) pathway which is involved in cardiac remodeling process was activated in response to norepinephrine and was mitigated by bucindolol. In conclusion, bucindolol was able to modulate cardiac remodeling which is mediated by oxidative stress.

## 1. Introduction

Heart failure (HF) is a global pandemic, affecting nearly 26 million people worldwide [1]. Pathologic cardiac remodeling in response to hemodynamic overload and cardiac injury is associated with the activation of the neuroendocrine system and an increase in oxidative stress and inflammation eventually leading to cardiac dysfunction [2]. The activation of the neuroendocrine system stimulates intracellular signaling pathways causing hypertrophy and fibrosis, activation of metalloproteinases, and increase of oxidative stress leading to cell death by necrosis or apoptosis. This process is a key step in the development of maladaptive hypertrophy, initiated by an imbalance between proapoptotic and antiapoptotic proteins [3]. Thus, the heart progresses from a compensatory phase of adaptive hypertrophy to dilation with decreased performance [4]. Therefore, blockage of the sympathetic system has an important role in the attenuation of cardiac remodeling in response to injury [5, 6]. Signal transducer and activator of transcription 3 (STAT3) is an important transcriptional factor that regulates cell proliferation, apoptosis, and cardiac hypertrophy. Cardiac STAT3 levels are dynamic and act as a sensor of both acute and chronic stress to protect against oxidative damage or cell death signaling [7].

Despite the key beneficial role of autophagy in physiologic cardiac remodeling, excessive or insufficient autophagy has been reported to contribute to adverse cardiac remodeling [8]. Cardiac autophagy has been reported to play an important role in response to increased norepinephrine levels [9]. Under nutrient-rich conditions, the phosphoinositide 3-kinase (PI3K)/protein kinase B (Akt)/mammalian target of rapamycin (mTOR) signaling cascade contributes to inhibiting autophagy, whereas, under stress condition, inhibition of mTOR caused by 5′ adenosine monophosphate-activated protein kinase (AMPK) activates autophagy, causing the conversion of the microtubule-associated protein 1 light chain 3 (LC3-I) to LC3-II, essential for the formation of autophagosomes [10].

Bucindolol is a nonselective *β*-adrenergic blocker (*β*1- and *β*2-blocker) with vasodilatory properties by *α*1-antagonism [11] and mild sympatholytic effects [12]. We have previously demonstrated that bucindolol treatment improved right ventricle (RV) systolic function; reduced RV pleomorphism, necrosis, and fibrosis; decreased infiltration of inflammatory cells; and decreased the sympathetic drive and balance and myocardial burden in rats with pulmonary arterial hypertension induced by monocrotaline [13]. Furthermore, bucindolol has also been reported to improve left ventricular systolic and diastolic function in humans [14].

Despite evidence showing cardiovascular benefits of bucindolol, its exact role in cardiac remodeling is still not fully known. In the current study, we explored the role of bucindolol in the signaling for hypertrophy/cell survival and apoptosis pathways in H9c2 cardiac cells exposed to norepinephrine.

## 2. Materials and Methods

### 2.1. Cell Culture

Rat cardiac cells (H9c2) (American Type Culture Collection, Manassas, VA, USA) were cultivated in Dulbecco's Modified Eagle's Medium (DMEM) (Sigma-Aldrich, St. Louis, MO, USA) supplemented with 10% (*v*/*v*) Fetal Bovine Serum (FBS) (HyClone Laboratories, Pittsburgh, PA, USA) and 1% antibiotic-antimycotic (100X) (10,000 U/ml of penicillin, streptomycin 10,000 *μ*g/ml of streptomycin, and 25 *μ*g/ml of Gibco amphotericin B) (Gibco/Life Technologies, Carlsbad, CA, USA) and incubated at 37°C with 5% CO_2_ and 100% humidity. To maintain the cell culture, medium was changed every 2 to 3 days and cells were passaged when they reached 75-80% confluence. Cells were seeded at 1 × 10^4^ cells/well for the MTT assay, 1 × 10^5^ cells/ml for analysis on the flow cytometry, and 1 × 10^6^ cells/T-75 flask for Western blotting.

### 2.2. Treatment Conditions

Four treatment conditions were established: (1) H9c2 cells that were not exposed to norepinephrine or bucindolol (CTL), (2) H9c2 cells exposed to norepinephrine (NE), (3) H9c2 cells incubated with bucindolol (CTL+BCD), and (4) H9c2 cells exposed to norepinephrine and bucindolol treatment (NE+BCD). The concentration of norepinephrine was determined by the MTT cell viability assay, where H9c2 cells were incubated with different concentrations of norepinephrine (2, 5, and 10 *μ*M) at two different time points (24 and 36 h). Based on the results, 10 *μ*M of norepinephrine for 24 h was used for subsequent experiments. Norepinephrine (Sigma-Aldrich, St. Louis, MO, USA) was diluted in 0.5 M HCl (0.03%), and incubation was done in FBS- and antibiotic-antimycotic-free culture medium. Subsequently, H9c2 cells were treated for 8 h with bucindolol (10 *μ*M) (Santa Cruz Biotechnology, Santa Cruz, CA, USA), prepared in 0.01% DMSO (Dimethyl Sulfoxide; Fisher Scientific, Ottawa, ON, CA).

### 2.3. Protein Expression

Western blot was used to analyze the protein expression of different signaling proteins. H9c2 cells were lysed in PathScan lysis buffer (25 mmol/l Tris pH = 7.5, 150 mmol/l NaCl, 1 mmol/l EDTA, 1% Triton-X 100), sodium fluoride, sodium orthovanadate, and protease inhibitors cocktail (Sigma-Aldrich, St. Louis, MO, USA), using the Qiagen Tissue Lyser. Samples were centrifuged at 8000 *g* for 10 minutes at 4°C and supernatants were collected, and the protein concentration was quantified using the DC protein assay kit (Bio-Rad Laboratories, Mississauga, ON, CA). Thirty micrograms of protein sample was subjected to SDS-page gel electrophoresis (7–15% according to the size of the proteins of interest) and electrophoretically transferred to a nitrocellulose membrane using a Trans-Blot apparatus (Bio-Rad Laboratories, Mississauga, ON, CA). The membrane was blocked with 5% nonfat milk in Tris-buffered saline (20 mM Tris, 137 mM NaCl) for 1 h at room temperature and then incubated overnight at 4°C with the following primary antibodies: 4-HNE (1 : 500); phospho-Akt (1 : 2000), #4060S; Akt (1 : 250), #9272; phospho-AMPK*α* (1 : 1000), #5256; AMPK*α* (1 : 250), #2603; phospho-mTOR (1 : 1000), #2971; mTOR (1 : 1000), #4517; LC3B-I/II (1 : 500), #2775; p70 S6 kinase (1 : 1000), #9202; PI3 kinase p85-*α* (pTyr^607^) (1 : 500); phospho-STAT3 (1 : 1000), #4113; and STAT3 (1 : 1000), #12640. All antibodies were purchased from Cell Signaling Technology (Danvers, MA, USA), except 4-HNE (ab46545; Abcam, Cambridge, MA, USA) and PI3K (ABS234; Millipore, Bedford, MA, USA). The bound primary antibodies were detected with anti-rabbit or anti-mouse horseradish peroxidase-conjugated secondary antibodies (1 : 1000–1 : 5000) and were normalized to *β*-actin (sc-47778; Santa Cruz Biotechnology, Santa Cruz, CA, USA). Chemiluminescence reaction was detected by the ChemiDoc XRS System, supported by Quantity One software version 4.6.5 (Bio-Rad Laboratories, Mississauga, ON, CA), which was analyzed by ImageJ software (Wayne Rasband, Research Services Branch, NIH, Bethesda, MD, USA).

### 2.4. Cell Death Assays

Apoptosis of H9c2 cells was measured by the presence of active caspase-3/7, detected by flow cytometry (FACSCalibur Flow Cytometer; BD Biosciences, San Jose, CA, USA) using the CaspaTag Caspase-3/7 *In Situ* Assay Kit (Chemicon International, Temecula, CA, USA), according to the manufacturer's instructions. Active caspase-3/7 positive cells were assessed on the basis of the fluorescence after incubation with Fluorochrome Inhibitors of Caspases (FLICA) reagent [15]. 10,000 events were acquired per trial, and the data were expressed as fluorescence intensity in the M2 area of the histogram. The activity of caspase-3/7 was expressed as the mean fluorescence intensity.

Necrosis was assessed via flow cytometric analysis of H9c2 cells incubated with propidium iodide (PI) solution (250 *μ*g/ml) (Millipore, Bedford, MA, USA) for 10 min. PI is membrane impermeable in live cells but binds to DNA of dead cells due to loss of cell membrane integrity, causing increase in the fluorescence. For the analysis, the percentage of PI positive cells was determined using a FACSCalibur Flow Cytometer (BD Biosciences, San Jose, CA, USA) (10,000 events) [16].

### 2.5. Reactive Oxygen Species (ROS) Assay

Intracellular reactive oxygen species (ROS) level was assessed by incubating H9c2 cells with CM-H_2_DCFDA (5-[and-6]-chloromethyl-2′,7′-dichlorodihydrofluorescein diacetate, acetyl ester) (Molecular Probes; Invitrogen, Carlsbad, CA, USA) for 30 min. After this incubation period, the dye solution was removed by aspiration, and the cells were trypsinized and centrifuged at 500×*g* for 5 minutes at 4°C. The samples were analyzed by flow cytometry. To analyze the quantity of ROS produced, we measured the fluorescence of CM-H_2_DCFDA at 488 nm on the FL1 channel of a BD FACSCalibur Flow Cytometer (BD Biosciences) supported by BD CellQuest Pro Software (BD Biosciences, San Jose, CA, USA) [15]. Fluorescence was expressed as mean fluorescence, after 10,000 events were acquired per trial.

### 2.6. Statistical Analysis

All statistical analyses were performed using SigmaPlot software (Version 12.0, Systat Software Inc., San Jose, USA). Normality was assessed using the Shapiro-Wilk test. Comparisons between groups were performed by two-way ANOVA, with a Tukey post hoc test. Differences between groups were considered statistically significant when *P* < 0.05.

## 3. Results

### 3.1. Signaling Protein Expression

**Figure 1:**
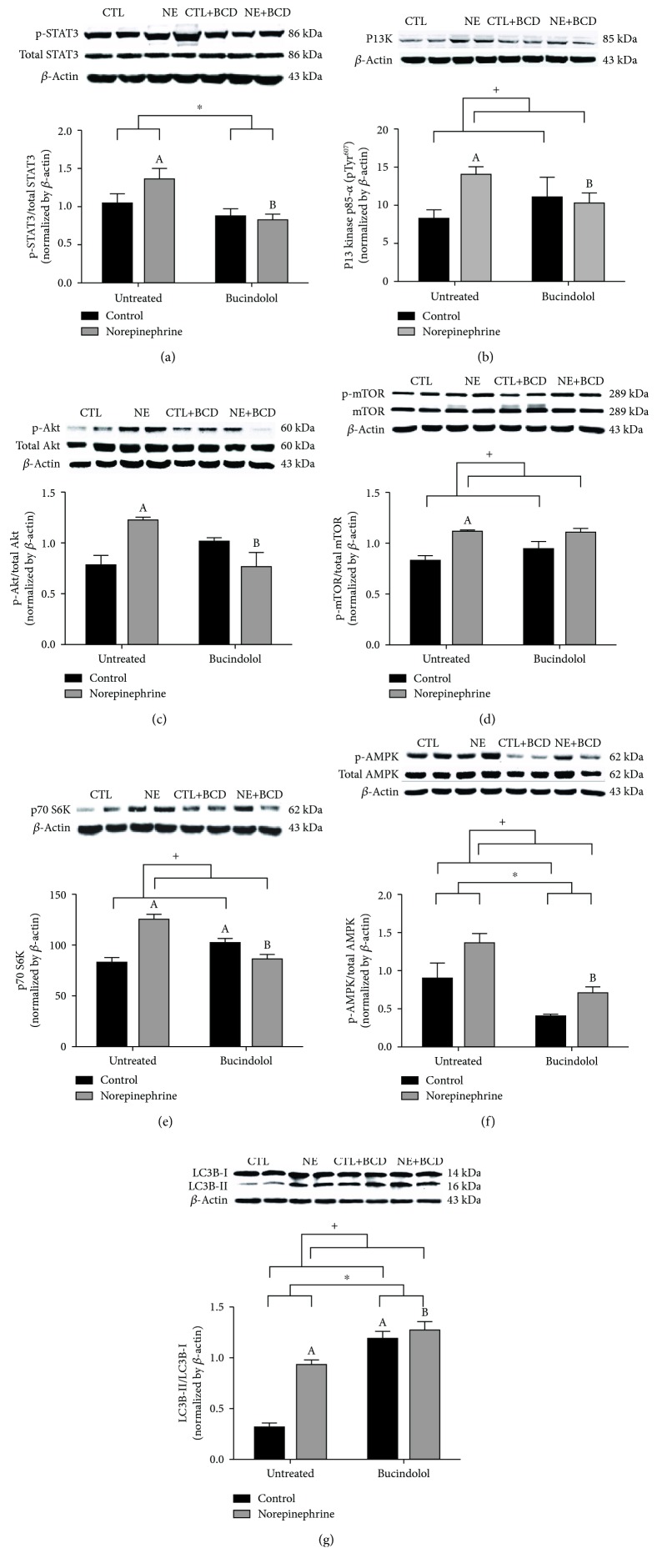
Western blot of proteins involved in cell survival and cell death signaling pathway. H9c2 cardiac cells were incubated with norepinephrine (10 *μ*M) during a period of 24 h, followed by 8 h of bucindolol (10 *μ*M) treatment. *β*-Actin was used for normalization. Immunoblot of p-STAT3/total-STAT3 (a), PI3K (b), p-Akt/total Akt (c), p-mTOR/mTOR (d), p70 S6K (e), p-AMPK/total AMPK (f), and LC3-II/LC3-I (g) was determined. Values were expressed as mean ± SD from 4 independent experiments. Two-way ANOVA followed by Tukey test: ^A^*P* < 0.05*vs.* Control Untreated (CTL), ^B^*P* < 0.05*vs.* Norepinephrine Untreated (NE), ^C^*P* < 0.05*vs.* Control+Bucindolol (CTL+BCD), ^+^*P* < 0.05 CTL and CTL+BCD *vs.* NE and Norepinephrine+Bucindolol (NE+BCD), and ^∗^*P* < 0.05 CTL+BCD and NE+BCD *vs.* CTL and NE.

To analyze the hypertrophy and cell survival signaling pathways, the immunoblot of the proteins STAT3, PI3K, Akt, mTOR, p70 S6K, and AMPK was performed. With regard to the transcription factor STAT3, there was a marked increase (32%) in its activity in the NE condition compared to CTL (Figure 1(a)). Bucindolol was effective in attenuating (39%) this increase in the NE+BCD condition. The activity of the protein kinases PI3K (Figure 1(b)) and Akt (Figure 1(c)) was found to be significantly increased (73% and 56%, respectively) in the NE condition in comparison to the CTL condition. Bucindolol was efficient in mitigating this increase, as NE+BCD demonstrated a 27% decrease in the PI3K expression and 38% decrease in Akt activity when compared to the NE condition. Moreover, there was an increase in mTOR activity (30%) in the NE condition as compared to the CTL condition, and bucindolol treatment did not alter mTOR activity (Figure 1(d)). However, the p70 S6 kinase (p70 S6K) expression was significantly higher (123%) in the NE condition when compared to the CTL condition, and the NE+BCD condition showed a decrease (32%) in the p70 S6K level in relation to the NE condition (Figure 1(e)). Similarly, we found an increase in AMPK activity (52%) in the NE condition when compared to the CTL condition (Figure 1(f)). Moreover, NE+BCD demonstrated a reduction of 48% in AMPK activity in relation to the NE condition.

The LC3-II/I ratio which reflects the autophagic activity was significantly upregulated in norepinephrine conditions (NE and NE+BCD) as compared to control conditions (CTL and CTL+BCD). Furthermore, the NE+BCD condition showed an increase of 35% in this ratio when compared to the NE condition (Figure 1(g)).

### 3.2. Cell Death

**Figure 2:**
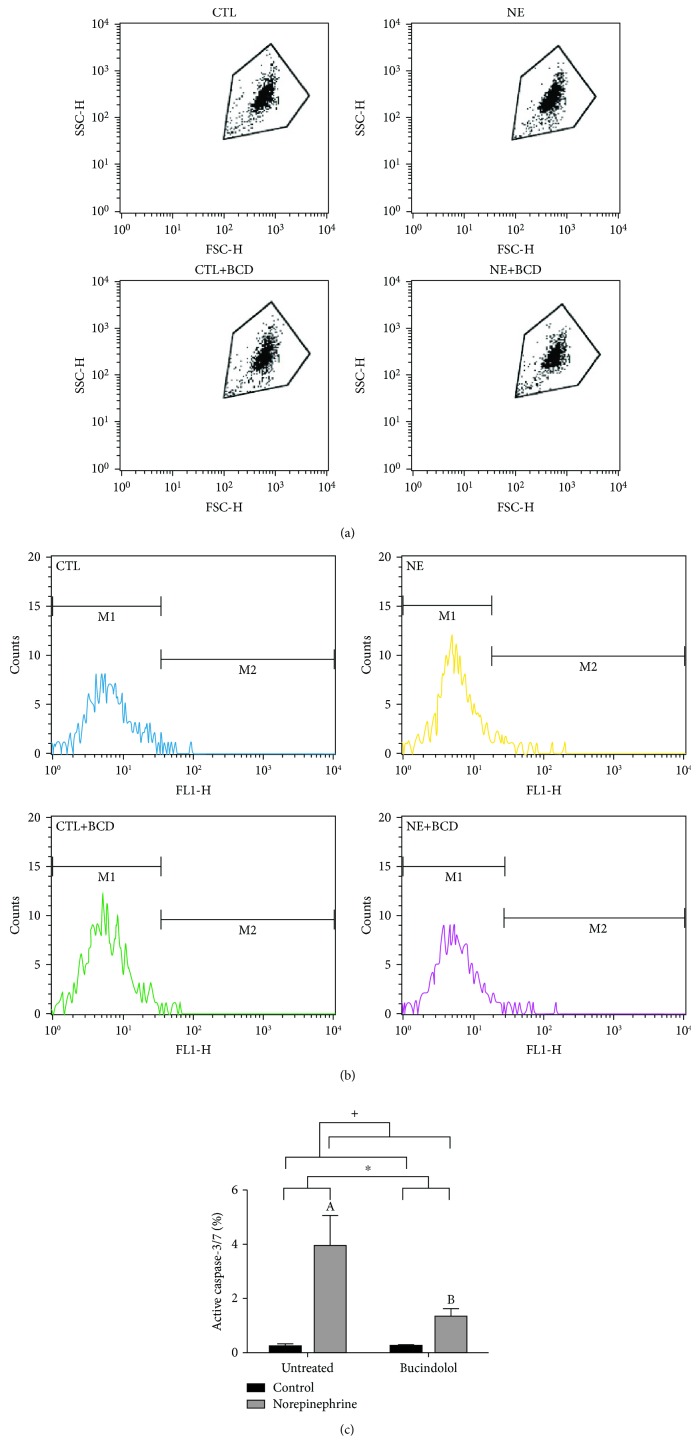
Active caspase 3/7 was determined in H9c2 cardiac cells incubated with norepinephrine (10 *μ*M) during a period of 24 h, followed by 8 h of bucindolol (10 *μ*M) treatment (c). Representative dot plots indicating the cell populations of all groups (a). Representative histogram showing FL1-H fluorescence versus cell counts, indicating activated caspase-3/7 (M2 area) (b). Values expressed as mean ± SD from 3 independent experiments. Two-way ANOVA followed by Tukey: ^A^*P* < 0.05*vs.* Control Untreated (CTL), ^B^*P* < 0.05*vs.* Norepinephrine Untreated (NE), ^+^*P* < 0.05 CTL and Control+Bucindolol (CTL+BCD) *vs.* NE and Norepinephrine+Bucindolol (NE+BCD), and ^∗^*P* < 0.05 CTL+BCD and NE+BCD *vs.* CTL and NE.

**Figure 3:**
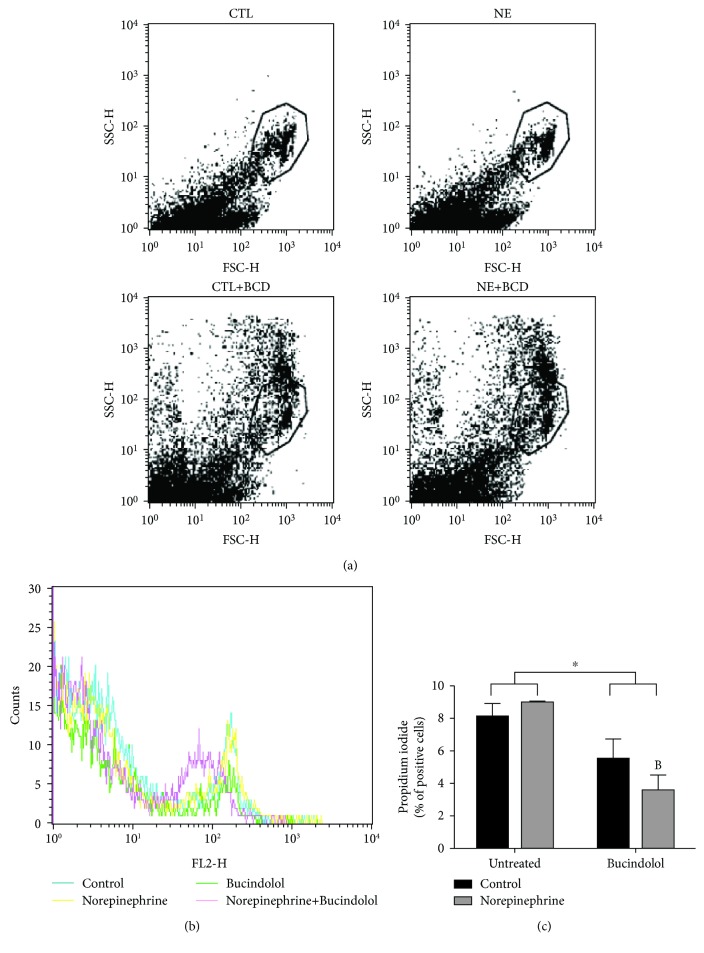
Cell death analysis by propidium iodide (PI) staining in H9c2 cardiac cells treated with norepinephrine (10 *μ*M) for 24 h, followed by 8 h of bucindolol (10 *μ*M). PI shows the percent of dead cells (c). Representative dot plots indicating the cell populations of all groups (a). Representative histogram indicating FL-2 fluorescence versus cell counts shown in (b). Values expressed as mean ± SD from 3-4 independent experiments (b). Two-way ANOVA followed by Tukey: ^B^*P* < 0.05*vs.* Norepinephrine Untreated (NE), ^∗^*P* < 0.05 Control+Bucindolol (CTL+BCD) and Norepineprine+Bucindolol (NE+BCD) *vs.* CTL and NE.

Cell death was assessed by measuring caspase3/7 activity and by PI staining. A significant increase in active caspase-3/7, a direct indicator of apoptosis, was evident in the NE condition in comparison to the CTL condition. Bucindolol was able to reduce (66%) this activity in the NE+BCD condition when compared to the NE condition (Figures 2(a) and 2(b)). In relation to PI staining, no significant differences were found between CTL and NE conditions. However, NE+BCD decreased (60%) the PI uptake when compared to the NE condition (Figures 3(a) and 3(b)).

### 3.3. Oxidative Stress

**Figure 4:**
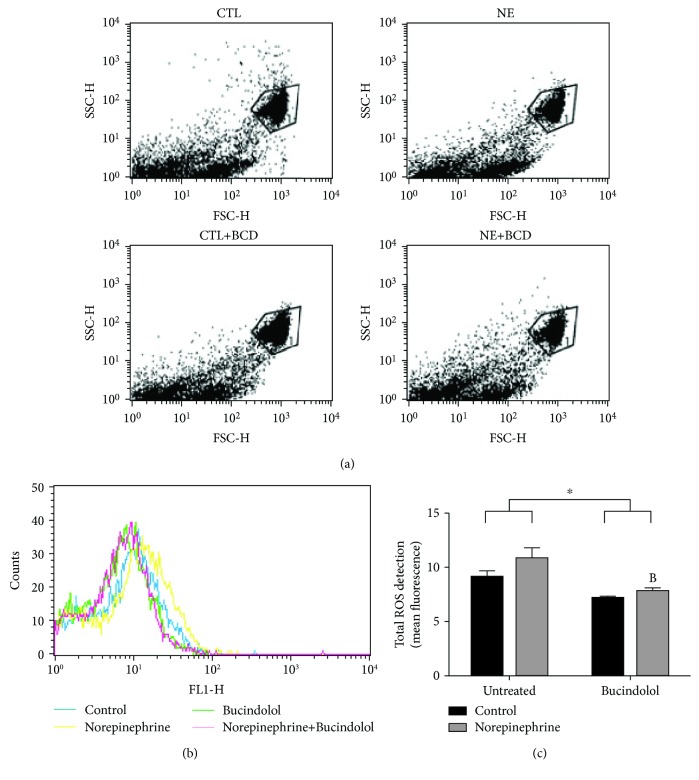
Oxidative stress analysis in H9c2 cardiac cells incubated with norepinephrine (10 *μ*M) for 24 hours, followed by 8 hours of bucindolol (10 *μ*M) treatment. ROS was determined by the CM-H_2_DCFDA assay and measured by flow cytometry (c). Representative dot plots indicating the cell populations of all groups (a). Representative histogram indicating cell counts of ROS levels versus FL1-H fluorescence (b). Values expressed as mean ± SD from 3 independent experiments. Two-way ANOVA followed by Tukey: ^B^*P* < 0.05*vs.* Norepinephrine Untreated (NE), ^∗^*P* < 0.05 Control+Bucindolol (CTL+BCD) and Norepineprine+Bucindolol (NE+BCD) *vs.* Control Untreated (CTL) and NE.

Intracellular ROS measured by the DCFH-DA assay are shown in Figures 4(a) and 4(b). No significant difference in oxidative stress was observed between CTL and NE conditions. However, the NE+BCD condition demonstrated a significant reduction (34%) of the ROS in relation to the NE condition.

**Figure 5:**
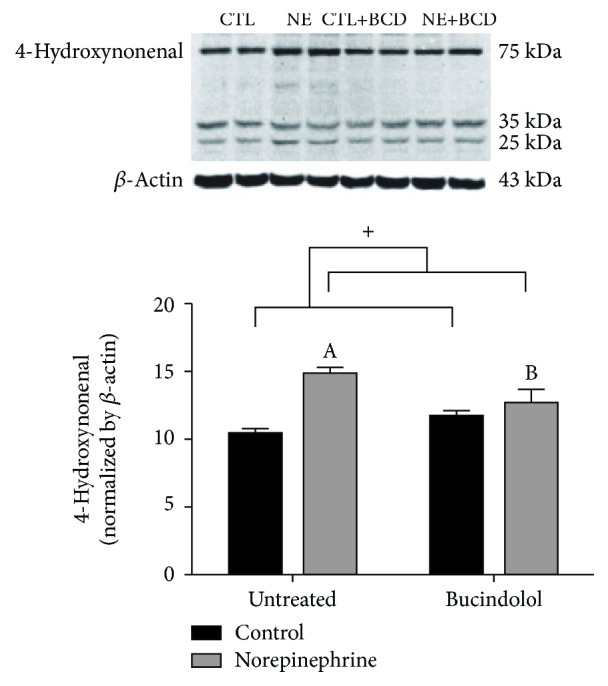
Lipid peroxidation measured by determination of relative protein levels of 4-hydroxynonenal (4-HNE) adducts in H9C2 cardiac cells incubated with norepinephrine (10 *μ*M) for 24 h, followed by 8 h of bucindolol (10 *μ*M) treatment. *β*-Actin was used for normalization. Values expressed as mean ± SD from 3-4 independent experiments. Two-way ANOVA followed by Tukey: ^A^*P* < 0.05 vs. Control Untreated (CTL), ^B^*P* < 0.05*vs.* Norepinephrine Untreated (NE), ^+^*P* < 0.05 CTL and Control+Bucindolol (CTL+BCD) *vs.* NE and Norepinephrine+Bucindolol (NE+BCD).

Furthermore, bucindolol was effective in mitigating the increase in lipid peroxidation caused by norepinephrine, as the NE condition showed an increase (44%) in the 4-HNE expression when compared to the CTL condition and the NE+BCD condition demonstrated a decrease (20%) in the protein expression in relation to the NE condition (Figure 5).

## 4. Discussion

In the present study, we have demonstrated that bucindolol, a nonselective *β*-blocker/sympatholytic agent, modulated cardiac remodeling by attenuating the signaling for apoptosis in an *in vitro* model of norepinephrine-induced cardiac damage, and this was associated with reduced oxidative stress.

Chronic stress results in prolonged cardiac exposure to norepinephrine, which through the activation of the *α*- and *β*-adrenergic receptors induces changes in cardiac function and structure, such as hypertrophy, which is an initial step of cardiac remodeling and eventually triggering cell death which represents the initial stage of heart failure [17]. Thus, the *in vitro* model of norepinephrine-stimulated cardiomyocytes is relevant and appropriate for assessing the cardioprotective potential of drugs against damage caused by the overactivation of the sympathetic nervous system [18].

Based on cell viability data (not shown), we chose 10 *μ*M norepinephrine for 24 h in the presence and absence of 10 *μ*M of bucindolol for 8 hours to investigate the effect of bucindolol on markers of apoptosis, cell survival, and oxidative stress.

The activation of adrenergic receptor induced by norepinephrine is a primary mediator of cardiac hypertrophy. STAT3 is an important transcription factor that regulates cell differentiation, proliferation, and apoptosis [19] and is known to be directly activated in cardiomyocytes by *β*-adrenergic agonists [20]. Kishore and Verma have reported that STAT3 signaling is activated during physiological hypertrophy and inhibited under chronic pathological conditions [21]. Other studies have demonstrated that persistent STAT3 activation promotes uncontrolled growth and survival through altered gene expression [22], leading to pathologic cardiac hypertrophy [20]. Our results showed an increase in STAT3 activity in the NE condition, which was reversed in the NE+BCD condition by bucindolol treatment. Our study is the first to report that bucindolol decreases STAT3 activity. The adrenergic receptor-induced cardiac hypertrophy response usually involves the activation of members of the MAPK family as well as the PI3K/Akt pathway. The action of norepinephrine on the *α*-adrenergic receptor in the heart results in the activation of phospholipase C and formation of inositol triphosphate (IP3) and 1,2-diacylglycerol (DAG). Subsequently, DAG stimulates cytosolic protein kinases activity, resulting in increased protein synthesis and development of hypertrophy [23]. Through this signaling cascade, PI3K is activated, leading to recruitment and activation of Akt. PI3K/Akt signaling is essential for conferring cardioprotection in response to stimuli and it activates STAT3. Phosphorylated STAT3 is linked to the activation of Akt, and this pathway is involved in cell proliferation and cell growth. Numerous Akt targets are involved in cardiac remodeling, including mTOR. When activated by Akt, mTOR phosphorylates p70 S6K, which plays a key role in the stimulation of protein synthesis in the heart [24]. It is known that the activity of these pathways is involved in the progression of cardiac hypertrophy to heart failure [25]. In our study, we found an increase in the activity of PI3K, Akt, and mTOR in the NE condition, as well as an increased expression of the p70 S6K. Thus, our results suggest cardiac hypertrophy in the NE condition. Bucindolol treatment resulted in a decrease in the activity and expression of these hypertrophy markers. Similarly, Pönicke et al. have reported that in rat ventricular cardiomyocytes, 10 *μ*M of bucindolol inhibited noradrenaline-induced protein synthesis [26].

The PI3K/Akt/mTOR signaling pathway has an important role in regulating proliferation, cell growth, and transcription of genes related to antioxidants, apoptosis, and autophagy [25]. Cardiac autophagy plays an important in the turnover of damaged macromolecules and organelles, which is a key in maintaining cellular homeostasis [10]. Excessive autophagy is observed in the damaged cells, which may be a trigger for cardiac apoptosis. During autophagy, damaged organelles and proteins are degraded in the autolysosome [10, 27]. Autophagy is initiated by phosphorylation of AMPK, which inhibits mTOR [27]. This signaling pathway leads to the conversion of the cytosolic precursor LC3-I into LC3-II, which is essential for membrane extension, curvature, and closure of autophagosomes [10, 27]. In our study, the NE condition demonstrated upregulation of the AMPK/mTOR pathway and increased LC3-II/LC3-I ratio. Previous studies have reported that STAT3 inhibited autophagy via inhibition of oxidative stress [28]. Xu et al. also demonstrated a significant increase in AMPK activity in norepinephrine-treated H9c2 cells, caused by the stimulation of *α*1-adrenergic receptor [29]. In the NE+BCD condition, we observed an increase in mTOR activity and LC3-II/LC3-I ratio. Recent literature suggests that the persistent adrenergic hyperstimulation that triggers autophagy in cardiac cells could contribute to reducing the deleterious effects of norepinephrine, acting as a cardioprotective response against apoptosis [9, 30].

While limited adrenergic stimulation causes myocyte hypertrophy, adrenergic overdrive contributes to myocyte loss because of altered antiapoptotic Bcl-2 protein expression. Activation of the *β*2 receptor is responsible for the antiapoptotic action [6]. It is known that cardiomyocytes predominantly express *β*1, and thus, the hyperactivation of these receptors has an important role not only in ventricular remodeling but also in inducing apoptosis [31, 32]. It is known that the imbalance between Bax/Bcl-2 is responsible in changing the mitochondrial membrane potential, generating the opening of mitochondrial membrane pores, with subsequent activation of the caspases [14, 15]. A significant increase of the activation of effector caspases-3/7 was evident in the NE condition in relation to CTL and NE+BCD conditions. In healthy cells, the caspases reside in the cytosol and its activation is achieved by autoactivation, transactivation, or proteolysis [33]. We have demonstrated that norepinephrine results in apoptosis and bucindolol has antiapoptotic action, as the activation of the caspases-3/7 is its final target. To confirm the cardioprotective role of bucindolol in cell death pathways, PI assessment was performed. Bucindolol significantly reduced the number of dead cells in the NE+BCD condition compared to the NE condition.

A recent study reported that different concentrations of NE lead to either hypertrophic or apoptotic response which is associated with distinct pattern of ROS production [34]. Moreover, Saleem and Goswami demonstrated that the activation of cardiac adrenergic receptors by norepinephrine costimulated the generation of Nox2, leading to the production of superoxide anion [35]. Increase in oxidative stress leads to lipid peroxidation, which modify cell integrity through altering cell permeability [36]. 4-HNE is the stable by-product of lipid peroxidation causing changes in cell signaling, DNA damage, and inducing apoptosis by the activation of the caspases [37]. Moreover, the PI3K-mediated cell survival pathway is known to protect against 4-HNE-mediated oxidative damage [38].

Although we did not observe a significant increase in ROS levels in the NE-exposed cells as measured by CM-H_2_DCFDA (which is a general indicator of oxidative stress), there was a significant increase in the protein expression of 4-HNE (a more specific marker of oxidative stress) suggesting that NE may lead to a distinct repertoire of ROS depending on the dose and exposure time as suggested by a recent study by Thakur et al. [34]. Bucindolol was able to attenuate oxidative stress in the NE+BCD condition, suggesting that bucindolol may possess antioxidant potential.

This study has certain limitations. These experiments were performed with H9c2 cardiac cells which is a commonly used cell line for the *in vitro* studies. Thus, our observations cannot be fully extrapolated to the *in vivo* conditions. However, H9c2 cells have also been used previously by other investigators to study the cardiac effects of norepinephrine on redox signaling [34, 35, 39]. Secondly, we only measured the protein expression of LC3B-I and LC3B-II to assess autophagy. Employing other techniques to measure autophagy flux would have been ideal.

## 5. Conclusion

**Figure 6:**
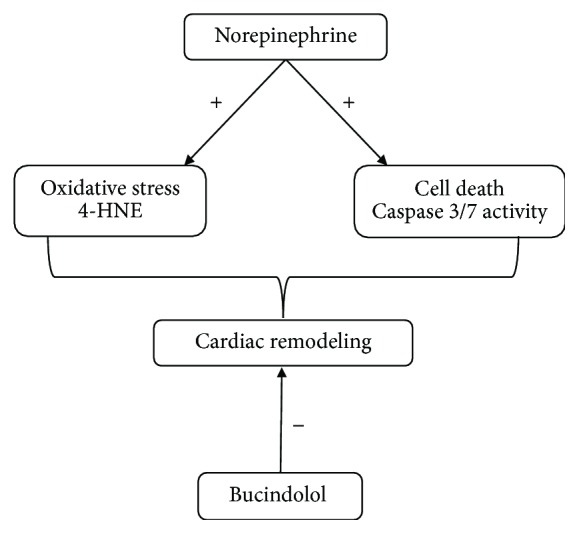
Bucindolol modulates norepinephrine-induced cardiac remodeling by attenuating oxidative stress and cell death.

Bucindolol treatment was able to prevent the increase in cardiac hypertrophy signaling and apoptosis, a characteristic of heart diseases due to sympathetic hyperactivity. The beneficial effects of bucindolol appeared to be due to the modulation of ROS generation and oxidative damage (Figure 6).

## Data Availability

The data used to support the findings of this study are included within the article.
